# Moving Beyond Indices: A Systematic Approach Integrating Food System Performance and Characteristics for Comprehensive Food Security Assessment

**DOI:** 10.3390/foods14101834

**Published:** 2025-05-21

**Authors:** Muna A. Al-Ansari, Hamad Nabeel, Galal M. Abdella, Tarek El Mekkawy

**Affiliations:** Department of Mechanical and Industrial Engineering, College of Engineering, Qatar University, Doha P.O. Box 2713, Qatar; 200356160@qu.edu.qa (M.A.A.-A.); gmg5005@qu.edu.qa (G.M.A.); tmekkawy@qu.edu.qa (T.E.M.)

**Keywords:** food security assessment, composite indicators, multiple regression analysis, two-step clustering

## Abstract

Food security indices are widely used to support decision making and provide a structured assessment of countries’ capacities to withstand global environmental and economic crises. However, these indices have inherent limitations, including potential biases in ranking and a lack of structural insights into food system dynamics. This study presents a systematic approach that combines elastic-net regression-based feature selection and two-step clustering to address some of these limitations and equip decision makers with structured procedures for making informed decisions and supporting food system management. The mathematical and operational procedures of the proposed approach were demonstrated through an illustrative example using the EIU dataset of 94 countries. The study investigated the sensitivity of composite indicators to extreme data points, relative weights, and dimensionality reduction. After applying elastic-net regression, 15 indicators were selected for Model 1 (M1) and 9 for Model 2 (M2) from an initial set of 25 indicators. Subsequently, two-step clustering grouped the countries into four distinct clusters, reflecting combinations of food system characteristics and income levels. The results demonstrate that countries with industrialized, consolidated food systems and high per capita income tend to exhibit greater food security. Conversely, countries with rural or traditional food systems and low-income levels are more vulnerable to food insecurity. By incorporating statistical rigor and empirical structure discovery, this methodology addresses key limitations of existing indices. It provides an adaptive, transparent framework that informs targeted policy by linking the structural characteristics of food systems to tangible food security outcomes.

## 1. Introduction

Food security refers to a state in which all individuals, at all times, have reliable physical, social, and economic access to sufficient, safe, and nutritious food that satisfies their dietary requirements and food preferences, supporting an active and healthy lifestyle [1]. It is a multidimensional concept that plays a vital role in the well-being and survival of humanity [2,3,4]. The four primary pillars of food security are availability, accessibility, stability, and utilization. Food availability refers to the physical presence of sufficient food, whether through domestic production or a reliable external supply. This availability depends on factors such as storage capacity, transport infrastructure, and the integration of local or national markets [5]. Food access pertains to a household’s ability to obtain sufficient and nutritious food through self-sufficiency, purchases, or food aid [6]. It is important to note that food may be available but not necessarily accessible to all households. Food stability is crucial for food security, as it reflects the system’s resilience and capacity to maintain a consistent food supply amidst shocks or disruptions to the local or global economy [7,8]. Lastly, food utilization examines individuals’ ability to use accessible food effectively. This can be achieved by ensuring an appropriate diet, safe drinking water, sanitation, and health care that meet nutritional and physiological needs. Thus, assessing food security comprehensively requires capturing these four dimensions, yet many existing indices struggle to reflect all pillars adequately.

Despite ongoing efforts by international institutions, global food systems face significant threats. Climate change poses a major challenge, disrupting agricultural productivity, water availability, and the overall stability of food systems. Rising temperatures, changing precipitation patterns, and more frequent extreme weather events reduce crop yields, alter growing seasons, and increase pest and disease pressures, compromising food availability and access [9,10,11]. Water scarcity is another critical concern, intensified by increasing global demand due to population growth, shifting consumption patterns, and energy production needs [12,13]. Recent data from the UN World Water Development Report (2025) indicate that agriculture still accounts for approximately 72% of global freshwater withdrawals, highlighting water’s pivotal role in ensuring food security. Insufficient access to water constrains crop and livestock productivity and impairs food availability, stability, and utilization.

According to the 2023 State of Food Security and Nutrition in the World (SOFI) report, 735 million people experienced chronic hunger in 2022, a significant increase from 613 million in 2019. Projections indicate that nearly 600 million people may remain chronically undernourished by 2030, threatening the achievement of Sustainable Development Goal 2, i.e., zero hunger. Additional environmental stressors, such as air pollution and soil degradation, further jeopardize food security. These factors affect agriculture, livestock, and aquaculture by altering pest dynamics, diminishing crop yields, and degrading soil fertility through erosion and overgrazing [14,15]. These pressures are compounded by the socioeconomic aftermath of the COVID-19 pandemic, which disrupted food supply chains, drove up food prices, and disproportionately impacted low- and middle-income families [16,17,18]. Addressing these challenges requires effective policy and governance frameworks; however, many countries face institutional and regulatory barriers that limit the implementation of sustainable and resilient food systems [19,20].

Several initiatives have been developed and implemented worldwide to eliminate hunger and malnutrition at local and global levels. The growing focus on these efforts highlighted the need for appropriate tools to help provide a condensed view of progress and make proactive adjustments to ensure the fulfillment of the strategic goals of these initiatives. Food security assessment is increasingly recognized as crucial for making informed decisions to mitigate risks and provide adequate food security amid various threats [21,22,23,24]. This assessment helps understand the impacts of deteriorating living conditions on food security and evaluates food safety and nutritional quality, especially for vulnerable populations [25]. Various methods are employed for food security assessment, including expert-based surveys, technical reports, and indicators like anthropometry, which evaluates nutritional status through physical measures [26], and the Food Insecurity Experience Scale (FIES), which measures food security based on personal experiences [27].

Notable reports such as the Global Nutrition Report (GNR) and the State of Food Security and Nutrition (SOFI) report provide comprehensive insights into global food and nutrition issues [22]. Recently, there has been a notable trend in using composite indicators to assess the outcomes of several food initiatives. These indicators are widely known for making food system performance more understandable and interpretable by combining different indicators (or factors) into a single index or score [21,22,23,24]. The Global Hunger Index (GHI) assesses hunger through indicators like malnutrition and child mortality [28], while the Global Food Security Index (GFSI) evaluates food security across over 100 countries using affordability, availability, and quality metrics, with recent updates including natural resources and resilience as indicators [29].

Despite the widespread use of composite indices like the GFSI, GHI, and FIES, several methodological limitations undermine their effectiveness. Chief among these are subjective weighting schemes, limited incorporation of outcome-based indicators, and lack of contextual benchmarking. For instance, while the GFSI provides broad insights into food system performance, it often fails to reflect populations’ actual consumption or nutritional outcomes [29,30]. Similarly, subjective weighting schemes usually rely on expert judgment, which can introduce bias, lack transparency, and reduce reproducibility in index construction [31]. These shortcomings reduce the reliability and policy relevance of food security assessments. To address these gaps, this study introduces a structured, data-driven framework that combines an input–output model, elastic-net regression, and a two-stage clustering method. The proposed approach enhances the interpretability of composite indices by selecting the most relevant indicators, incorporates outcome-based measures to link resources with real-world impact, and facilitates context-aware benchmarking through cluster analysis. Together, these methodological innovations aim to improve food security assessments’ robustness, clarity, and policy relevance.

## 2. Literature Review

Food security assessment utilizes various methods such as expert-based surveys, annual technical reports, and food security indicators. Recently, there has been a growing trend towards employing composite indicators to evaluate the outcomes of food initiatives. These composite indicators are appreciated for their ability to simplify and enhance the interpretability of food system performance by integrating multiple indicators into a single index or score [21,22,23,24]. Three widely recognized food security indicators are discussed below.

The Global Food Security Index (GFSI), developed by the Economist Intelligence Unit (EIU) and sponsored by Corteva Agriscience in 2012, is a widely known measure of food security for over 100 countries. The GFSI initially involved three categories namely affordability, availability, and food quality and safety [29,32]. In 2020, the GFSI included a new category, natural resources and resilience, which was later renamed “Sustainability and Adaptation” in the 2022 GFSI Report. This category assesses the likelihood of natural disruptions and evaluates the ability of the system to adapt to their potential impacts and consequences. Several studies have assessed the procedures for computing the GFSI over the years (see [29,32,33]). These studies have revealed two main limitations. These are the use of subjective weighting methods and the lack of connecting the GFSI with indicators associated with food system outcomes of the associated countries, such as food consumption, food production, food supply, poverty or nutritional policies, and the population’s nutritional status [29,30].

The Food Insecurity Experience Scale (FIES), developed by the FAO, is another measure of food security. The FIES assesses food security at the household or individual level by capturing personal experiences [27]. The FIES utilizes an eight-question-based survey module, FIES-SM, to assess the difficulty individuals or households usually encounter in reaching adequate amounts of food and nutrition. While the FIES offers valuable insights into the lived experiences of food insecurity, it has also faced criticism. Short reference periods, particularly the 30-day version, can yield severity assessments that differ substantially from the 12-month reference frame, complicating global comparisons [34]. Additionally, the FIES’s focus on individual experiences may oversimplify the complexity of food insecurity by neglecting broader systemic factors such as sustainability, structural inequality, or democratic agency within food systems [35].

The Global Hunger Index (GHI), developed by the International Food Policy Research Institute (IFPRI), measures global hunger status. The GHI utilizes a combination of four indicators to determine caloric deficiency and malnutrition, namely malnutrition, child undernutrition, stunting and wasting in children, and child mortality. The GHI scores are calculated through a three-step process. The four indicators are first given standardized scores from 0 to 100, where 0 indicates “no hunger” and 100 indicates “severe hunger”. Following this step, to calculate the Global Hunger Index (GHI) for each country, the standardized scores of the four key indicators are aggregated, with equal weights assigned to each indicator [28,32]. The data on the GHI are collected from the U.N. database and other multilateral agencies (for further details, visit https://www.globalhungerindex.org)**.** Despite its widespread use, the GHI has been criticized for its methodology and conceptual framing. One notable concern is its narrow focus on caloric intake and child health outcomes, which may overlook broader dimensions of food insecurity, such as dietary quality, food access, and socioeconomic determinants [35].

While indices like the GFSI, FIES, and GHI offer useful snapshots of food security conditions, their structural and methodological limitations motivate the need for enhanced, data-driven approaches that better capture food system complexity.

### Existing Weighting Methods

The weights are crucial to ensure the accuracy and credibility of the food security index. These weights refer to the importance of each indicator (or factor) to the overall score of the composite index. Several methods for estimating relative weights under various contexts have been conducted over the last years (for further reading, see [36,37,38,39]). Equal weighting is very popular in business and finance applications [40]. The equal weighting assigns equal importance to all indicators. However, several studies have criticized equal weighting for its shortcomings in accommodating double counting, which arises when two or more indicators measure the same aspect within the context of the GFSI calculations [41,42]. Common examples of methods used to address the issue of double counting in situations like these are the Analytical Hierarchy Process (AHP) and the Technique for Order of Preference by Similarity to the Ideal Solution (TOPSIS). These methods help mitigate the duplication of indicators measuring the same aspect within the GFSI framework. Over the years, some researchers have found that using expert judgments to assign weights, which is frequent in practice, may affect the credibility of the composite index [42,43]. While expert judgment remains a common approach, it is susceptible to cognitive and group-level biases that can distort weight assignments. For instance, cognitive biases such as overconfidence, anchoring, and availability can influence experts’ decisions, leading to inconsistent or suboptimal outcomes [44,45,46]. Additionally, when experts work in groups, social dynamics like groupthink or social loafing may further undermine the objectivity of the process [45]. Beyond biases, challenges in calibrating expert input and the inherent uncertainty of subjective weights add further complexity, making cross-study comparisons difficult and often unreliable [47,48]. Moreover, over-reliance on experts assumes complete domain knowledge, which may not be realistic in multifaceted contexts such as sustainability assessment [49]. Other methods, such as Principal Component Analysis (PCA), Data Envelope Analysis (DEA), and Factor Analysis (FA), have also been utilized for estimating relative weights in various fields (see [50,51]). PCA is mainly known for its efficiency in finding a new set of components (features), called principal components (PCs), that capture the most significant variability in the original dataset. Mainly, it reduces the dimension of the original dataset (see [50,52]). However, despite its popularity, the new component may not have a clear real-world interpretation [53,54].

In practice, especially under high-dimensional settings, food security indicators are more likely to exhibit high correlation levels, a condition known as multicollinearity. This statistical phenomenon occurs when some indicators are interrelated, potentially influencing the generation of weights and leading to instability in regression model estimates. As a result, the robustness of the constructed composite indicators may be compromised. While multicollinearity is more pronounced in high-dimensional datasets, it can also arise in lower-dimensional contexts [55,56]. Classical regression methods have shown limited effectiveness in addressing the multicollinearity phenomenon. On the other hand, penalization regression methods, such as ridge, least absolute shrinkage and selection operator (lasso), and elastic net, are known for effectively managing multicollinearity and sparsity [38,57,58]. These methods stabilize model estimates by introducing regularization penalties, with elastic net offering the dual advantage of variable selection and multicollinearity management.

In parallel, several statistical and machine learning methods have also been explored in the context of food security. While widely adopted, econometric models often rely on historical assumptions and fail to adapt to the dynamic nature of food systems [59]. Machine learning models and neural networks offer strong predictive capabilities but typically demand extensive, high-quality datasets and often lack transparency [60,61]. Bayesian spatio-temporal models effectively capture spatial and temporal dependencies but require significant computational resources and careful specification [62]. Panel quantile regression captures heterogeneous effects but needs extensive panel data [63], while small-area estimation improves subnational insights but is sensitive to model and data assumptions [64,65]. Nonparametric methods like random forests can model nonlinear relationships but are less interpretable [66]. Similarly, although useful in benchmarking efficiency, data envelopment analysis (DEA) is susceptible to input selection and lacks mechanisms for handling noise or measurement error [67].

Despite the increasing popularity of these methods, the application of penalized regression models in food security, especially for developing composite indices, remains limited and is still an emerging area of research [38,68,69,70,71]. In this context, elastic-net regression offers a particularly suitable approach due to its ability to integrate the benefits of both ridge and lasso regularization, thereby enabling robust estimation while performing automatic feature selection. This is especially beneficial in identifying the most relevant food security indicators and mitigating the risk of overfitting, which is common in high-dimensional data scenarios [72].

Furthermore, existing food security indices predominantly rely on input-based models that focus on resource availability, failing to connect these inputs with the actual outputs of food systems. Consequently, these models do not adequately capture how efficiently resources are utilized, thus providing an incomplete picture of food security. Moreover, interpreting composite indices becomes challenging for stakeholders and decision makers, especially when multiple indicators are involved, leading to difficulties in identifying specific areas of strength and weakness within food systems. Moreover, the prevalent use of single-score-based rankings in food security assessments often overlooks crucial contextual information, such as geographical location, food system typology, and economic state, resulting in the loss of valuable insights necessary for realistic benchmarking and effective solution development.

To address these limitations, this study introduces a structured approach to promote the current practices of utilizing composite indices to assess food security locally and globally. This approach uses two well-established methods, feature selection-based regression and clustering methods, which provide deep insights into food security issues and lead to efficient resource allocation. The list below explains the significance of this approach’s structured design compared to its counterparts.

1. Food security indices using input-based models, focusing on resource availability, do not relate the composite index to the food system outputs. In other words, these models do not clearly show how efficiently these resources are utilized. To overcome this limitation, this study introduces an input–output model that incorporates nutritional status as an output indicator. This output indicator is important to measure people’s accessibility to enough nutrition and the availability of their daily dietary needs.
2. Interpreting composite indices is critical to highlighting specific areas of strength and weakness in food systems. However, stakeholders and decision-makers may encounter serious difficulties interpreting the results when many indicators are involved. To mitigate this, elastic-net regression is employed to reduce the dimension of the impact matrix and include only the indicators relevant to the food system’s performance. This step will help understand the contribution of each indicator (or factor) and effectively address the root causes of food insecurity.
3. Using single-score-based rankings in food security assessments often disregards critical contextual factors, such as geographic location, food system typology, economic development levels, etc. The involvement of such characteristics is essential for conducting realistic benchmarking and developing short- and long-term solutions. This study proposes a two-stage clustering method to enhance food security analysis. First, entities (countries) are grouped based on their overall food security score. Following this, each cluster is further analyzed through a second round of clustering, considering key food system characteristics like typology and economic status. By creating more nuanced groupings, this approach aims to provide stakeholders with actionable insights for targeted interventions instead of relying solely on a single-score list.

The rest of this paper is organized as follows: Section 3 highlights the elastic-net regression method as a key element of the study’s methodology and outlines the proposed approach. Section 4 reports the illustrative example used to assess the applicability of the new approach. Section 5 reports the results of implementing the two-step clustering approach. Section 6 is dedicated to discussing the main findings and conclusions extracted from the case study. In addition, this section provides recommendations and suggests future research directions.

## 3. Methodology

**Figure 1:**
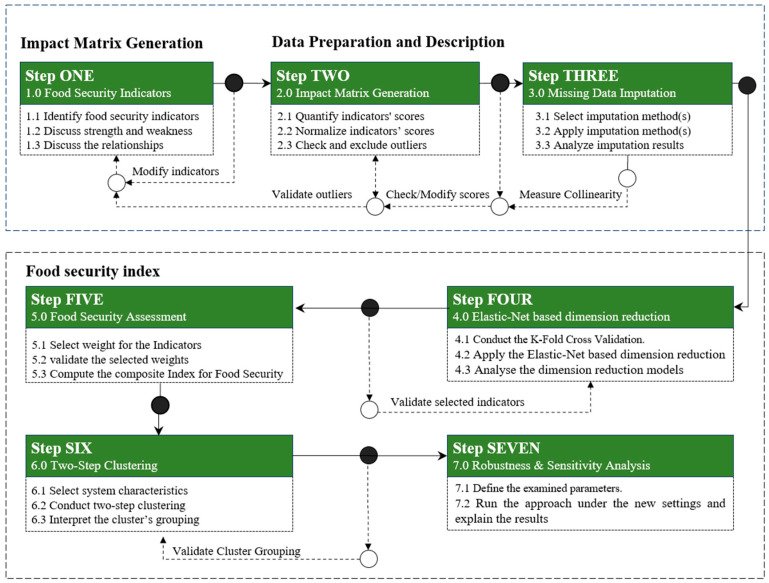
The outlines of the proposed approach.

Addressing methodological gaps identified in the literature, this study introduces a new approach combining two well-known methods: feature selection and two-step clustering (see Figure 1). The proposed methodology addresses several limitations of traditional input-based models that focus solely on resource availability without linking the composite index to food system outputs. By incorporating an input–output model, the population’s nutritional status is added as a crucial output indicator, representing the accessibility and availability of daily dietary needs. Furthermore, to improve the interpretability of composite indices, elastic-net regression is employed to reduce the dimensionality of the impact matrix, retaining only the most relevant indicators for food system performance. This technique was chosen for its ability to handle multicollinearity, a common issue in datasets with correlated predictors, ensuring that the selected indicators remain statistically independent. Unlike alternative methods such as Principal Component Analysis (PCA) or ridge regression, elastic-net regression allows for the simultaneous selection of a subset of predictors while maintaining model interpretability, a key feature for understanding the relationships between indicators in the food security context. This step aids in identifying and addressing the root causes of food insecurity.

Additionally, the study critiques the conventional single-score-based ranking system, which often overlooks valuable information related to specific characteristics of entities, such as geographical location, food system typology, and economic state. To overcome this, a two-stage clustering method is proposed: first, group entities based on their overall food security scores, and then, further, each cluster is analyzed through a second round of clustering that considers key food system characteristics. This nuanced clustering approach aims to provide stakeholders with actionable insights for targeted interventions, offering a more comprehensive understanding of food security dynamics than traditional methods.

The proposed approach consists of seven steps that were sequenced in a way to ensure a logical flow of computations and analysis as follows:

**Steps 1–2**: These initial steps involve identifying relevant food security indicators, assessing their impact within each category, and then integrating them to construct the initial impact matrix.

**Step 3**: Once the matrix is developed, its structure is examined for potential missing data. In consultation with domain experts, an appropriate data imputation method is selected to address any gaps and ensure data reliability.

**Step 4**: This step plays a pivotal role in the methodology by applying elastic-net regression to identify the most influential indicators contributing to food security performance. To enhance the accuracy and robustness of the results, the elastic-net regression is fine-tuned using a widely accepted optimization technique, i.e., k-fold cross-validation. A detailed explanation of this method is provided later in this section.

**Step 5**: In this step, various weighting methods are explored, and the most suitable approach is selected to compute relative weights for the indicators retained in the reduced impact matrix.

**Step 6**: Once each entity’s food security score is calculated, two-step clustering is applied to examine the relationships between food security performance and selected food system characteristics.

**Step 7**: A sensitivity analysis is conducted to assess how variations in key methodological choices, such as data imputation and dimensionality reduction, affect the performance of the composite food security indicator. This step helps identify the composite indicator’s robustness and highlights how methodological choices may lead to alternative rankings or interpretations, informing more transparent and evidence-based decision-making.

Elastic-net regression assumes linearity in the relationship between the predictors and the outcome variable, a common assumption in composite index modeling, where relationships are typically additive. However, while this method assumes linearity, non-linearities may be present but are not explicitly modeled in this framework. Additionally, elastic-net regression requires the careful handling of outliers, as extreme values can impact the regression results. To mitigate this, outlier detection and treatment are performed before the application of the model. A key advantage of the elastic net is its ability to handle multicollinearity by combining the strengths of both ridge and lasso, ensuring that correlated predictors do not overly influence the model.

To introduce the elastic-net regression, we begin with the generalized linear model, which describes the relationship between the dependent variable, y, and a set of independent variables, x.

(1) $$y_{i}=\beta_{0}+\sum_{j=1}^{p}\beta_{j}x_{ij}+\varepsilon_{i}\;\;\;;\;\;\;\;\;\;\;i=1,2,\ldots$$

where $y_{i}$ is the response variable, and $\beta_{j}{\in R}^{p}$ are the coefficients of the regression model associated with the jth independent variable. The term $\varepsilon_{i}$ represents the error and is assumed to be normally distributed. The regression coefficients can be found using the following optimization problem [73,74,75]:

(2) β^net=y−Xβ Ty−Xβ+Pλ(β) 

where $P_{\lambda }(\beta )$ is the elastic-net penalty function. Equation (2) can be also written as follows:

(3) β^net=y−Xβ Ty−Xβ+λ1∑j=1pβj+λ2∑j=1pβj2

In Equation (3), the term $\lambda_{1}\sum_{j=1}^{p}\left|\beta_{j}\right|$ corresponds to the $l_{1}$(lasso) penalty, which promotes sparsity, and $\lambda_{2}\sum_{j=1}^{p}\beta_{j}^{2}$ corresponds to the $l_{2}$(ridge) penalty, which addresses multicollinearity [57]. The tuning parameters $\lambda_{1}\;\geq \;0$ and $\lambda_{2\;}\;\geq \;0$ control the strength of the respective penalties. Notably, the elastic net has the advantage over other penalization methods because it does not require n>p. However, the parameter λ controls the penalization’s strength. If λ is equal to zero, the regression problem in Equation (3) will become a general linear regression.

Numerous techniques for estimating the value of λ are described in the literature. The k-fold cross-validation (CV) technique is the most frequently used [75]. The initial dataset is divided randomly into k equal-sized subsets. Then, one subsample is used as a validation dataset, and the remaining datasets are used as training subsets. These procedures should be repeated k times. The k results can be averaged to obtain a single estimate. Each recommended value of $\lambda_{1}$ and $\lambda_{2}$ will be evaluated using the CV procedure. Finally, the most accurate value of λ is selected as the optimal for performing elastic-net regression.

## 4. Illustrative Example

This example details the implementation process for the new food security index. Guidelines and relevant remarks will be provided under each step.

### 4.1. Food Security Indicators

This study utilizes the GFSI framework developed by the Economist Intelligence Unit (EIU) and supported by Corteva Agriscience. The framework comprises four main categories: food affordability (six indicators), food availability (seven indicators), food quality and safety (five indicators), and natural resources and resilience (seven indicators). Table 1 presents the details on the specific indicators within each category. Detailed information regarding the indicators’ specific weights and units can be found on the GFSI website [76].

This study focuses on 94 countries selected from the GFSI framework, based on the availability of data on the “prevalence of undernourishment as a percentage of the population”. This indicator acts as the food system output (response variable) in the study.

### 4.2. Impact Matrix Generation

This study uses the 2019 GFSI dataset, which can be accessed on https://impact.economist.com/sustainability/project/food-security-index/ (accessed on 20 March 2022). To ensure meaningful comparability across all indicators, the dataset was normalized column-wise (i.e., per indicator) using the Min–Max (Feature Scaling) algorithm [77,78]. The Min–Max algorithm calculates the normalized scores using the following:

(4) $$x_{ij}^{norm}=a_{0}+\frac{\left(x_{ij}-M_{min,j}\right)\left(a_{1}-a_{0}\right)}{\left(M_{max,j}-M_{min,j}\right)};i=1,2,\ldots ,n,\;\;\;\;\;j=1,2,\;\ldots ,\;p\;$$

where $x_{ij}$ represents the original score of the ith country under the jth indicator, $M_{max,j}$ and $M_{min,j}$ represent the maximum and minimum scores of the jth indicator, n represents the number of countries (n = 94), and p represents the number of indicators (p = 25). The constants $a_{0}$ and $a_{1\;}\;$($a_{0}<a_{1}$) are predetermined min–max values for the range of $x_{ij}^{norm}$. The 2019 GFSI dataset is normalized using $a_{0}$ = 0 and $a_{1}$ = 100, such that the highest possible score is 100, while the smallest possible score is 0.

The correlation analysis is necessary for understanding the inter-relationship between the indicators of the composite index; [29,71] have analyzed the correlation structure of the GFSI, considering the first three categories. Consequently, we will dedicate our analysis primarily to the fourth category, “Natural resources and resilience”. NR&R was first introduced into the GFSI in 2017 and was used as one of the main categories in 2020. This category consists of seven indicators, as shown in Table 1. The “Exposure” directly quantifies the impact of the crisis brought on by weather-related changes. This indicator may significantly impact countries in regions susceptible to frequent and severe climate changes and crises. The indicators “Water”, “Land”, and “Oceans, rivers, and lakes” describe the capacity of natural resources. It makes sense to include these three indicators, as they are essential for local and worldwide food producers and suppliers. Lastly, the “Political commitment to adoption” indicates the readiness for developing and executing effective food security solutions.

Pearson’s correlation coefficient (r) is widely used in practice for measuring correlation [52]. The r values range between −1 and 1, such that the higher the $r_{ij}$ value, the higher the correlation levels between the ith and jth indicators. When the ith and jth indicators are not significantly correlated, the $r_{ij}$ value will be near or equal to 0. The correlation analysis among the NR&R indicators will be based on the normalized 2019 GFSI dataset. The correlation coefficients for all possible indicator pairs are presented in Table 2.

The r values in Table 2 show that W, PCTA, and DS have the highest correlation levels (0.531, 0.535, and 0.803) with the 2019 GFSI scores. A two-sample *t*-test is applied to clarify the linearity between these indicators and the 2019 GFSI scores. The two-sample *t*-test is a statistical procedure used to determine if there is a significant difference between the means of two independent datasets. Two hypotheses will be examined against each other. These are H_0_:r = 0 versus H_1_: r ≠ 0, where r is the slope measure. The test is initiated by calculating the *p*-value. The decision in the *t*-test is made by comparing the *p*-value with the significance level (α), where α is a pre-defined threshold for rejecting the H_0_. If the *p*-value < α, we should reject hypothesis H_0_ and conclude that there is a linear relationship; otherwise, if the *p*-value ˃ α; we should accept hypothesis H_0_ and conclude that there is no linear relationship between the compared pairs of datasets.

The three pairs of indicators (W; GFSI), (PCTA; GFSI), and (DS; GFSI) were tested, and the *t*-test revealed *p*-values = 0.00001 when α= 0.05. Since the three *p*-values ˂ 0.05, we should reject H_0_ and conclude that there is sufficient evidence of a linear relationship between the 2019 GFSI scores and the tested indicators (W, PCTA, and DS). It is important to remember that this decision is limited to the case when α = 0.05. Other values of α ˂ 0.05 may reveal different decisions.

The pairwise correlations in Table 2 reveal that most indicators exhibit weak correlations with each other, with values ranging from −0.016 to 0.426. However, the same indicators exhibit slightly higher correlation values with the NR&R’s score. The linearity between the three top indicators, W, PCTA, and DS, has been examined using the two-sample t-test. Similar to the previous *t*-test results, the three pairs of indicators (W; NR&R), (PCTA; NR&R), and (DS; NR&R) revealed *p*-values = 0.00001 when α= 0.05. Hence, we can conclude that there is a significant linear relationship between these indicators and the NR&R’s scores.

However, despite these findings highlighting the importance of including these indicators in food security assessment, other data quality and availability factors should be considered. The following section discusses one of the common data issues, mainly outliers (or extremes), and examines their impact on the GFSI ranking scores.

### 4.3. Outliers Detection and Imputation

Data points that significantly deviate from most data points in the dataset are known as outliers. Outliers can disrupt the proposed approach in two critical ways: (1) outliers may significantly influence the results of the elastic-net regression, leading to improper selection of the most relevant indicators, and (2) outliers may lead to an inflation of the composite index, leading to misrepresentation of the actual contribution of indicators on the food security scores of the corresponding country.

Several outlier detection methods exist in the literature, such as the Interquartile Range (IQR), Z-scores, and winsorized methods. Each method offers unique advantages and limitations, with the optimal choice depending on the data characteristics and analysis goals. The winsorized method detects outliers and replaces them with less extreme values. The authors of [29] examined this method to substitute the outliers in the 2016 GFSI dataset. The study reported that the change in countries ranking with or without winsorized-based replacement is not significant.

To expand the research context in this regard, this study uses the Z-score method to identify potential outliers in the 2019 GFSI datasets. The outliers using the Z-score method are defined as data points whose Z-scores fall outside the Kth standard deviation. First, we compute the Z-score for each $x_{ij}^{norm}$ using $(x_{ij}^{norm}-\mu_{i})/\sigma_{j}$, where $\mu_{i}$, and $\sigma_{j}$ represent the mean and standard deviation of the jth indicator. Once the data have been converted, the new dataset of the jth indicator is assumed to follow the standardized normal distribution Z($\mu_{j}^{Z},\sigma_{j}^{Z})≅$ Z(0,1); then, each Z-score is associated with each data point, $x_{ij}^{Z}$, represents the distance from the center in terms of $\sigma_{j}^{norm}$. According to the Z-score approach, the outliers are the countries whose absolute Z-score value is higher than the threshold $K\sigma_{j}$, where $K\sigma_{j}$ = 3.

While the Z-score method with a threshold of ±3 is widely adopted in the literature for detecting outliers in normally distributed data, we acknowledge that it assumes approximate normality and may be sensitive to extreme values in skewed distributions. Alternative approaches, such as the Median Absolute Deviation (MAD) method, are more robust in the presence of skewed data or outliers, as they rely on the median rather than the mean. However, preliminary data exploration showed that most indicators followed approximately symmetric distributions after normalization, making the Z-score method a suitable choice for this study. Additionally, using a ±3 threshold ensures that only the most extreme values (less than 1% in a normal distribution) are flagged as outliers, aligning with related studies’ practices [79].

The Z-score test was applied to all the indicators, and the results showed that the 2019 GFSI dataset contains twelve outliers distributed across six indicators, accounting for 24% of the indicators. The CFC and AIT each involve three outliers, accounting for 50% of the outliers. However, there are two possible scenarios in practice for dealing with outliers. These are outliers’ exclusion or substitution. While excluding outliers is essential to have outlier-free datasets, it is important to carefully investigate how the number of excluded data points affects the credibility and applicability of the composite index.

Like [29], this study replaces the outliers with the nearest low/high non-outlier values. The calculations were performed, and the six indicators exhibited a pre- and post-replacement skewness as follows: CFC {−1.90, −1.74}, AIT{−1.61, −0.96}, FL{−1.61, −0.57}, MA{−1.00, −0.83}, E{−0.69, −0.47}, and W{1.45, 1.36}. The two-sample *t*-test was applied to statistically check the significance of differences between the Z-scores of the six indicators pre- and post-replacement. Let $\mu_{pre}$ and $\mu_{post}$ be the mean of the Z-scores of the pre- and post-replacements, respectively. Then, the two hypotheses of the *t*-test are set as H_0_$:\mu_{pre}-\mu_{post}=0$ and H_1_: $\mu_{pre}-\mu_{post}\neq 0$. Table A1 in Appendix A reports the settings and outputs of the *t*-test conducted using α = 0.05. However, since the *p*-value = 0.517 ˃ 0.05, we fail to reject H_0_ and conclude that the means of the pre- and post-replacement Z-scores are likely to have the same mean when α = 0.05.

For further investigation of the influence of the replacement method on the food security rank, we compared the original GFSI ranking with its counterpart using the nearest low/high non-outliers. As shown in Table 3, while 25% of countries experienced a change in rank after outlier imputation, most of these shifts were limited to ±1 position. This aligns with previous findings by [29], where the winsorization of GFSI indicators also led to minimal impact on rankings, with most countries shifting by only one or two positions. Based on these findings, we can roughly conclude that the imputation method utilized in this study has no significant effect on the 2019 GFSI ranking scores.

Like many others, the nearest low-/high-imputation method can be influenced by outlier characteristics such as the number, frequency, and distribution. Alternative approaches that account for these factors are often preferable. This is especially true in high-dimensional indicator settings, where traditional methods might encounter serious challenges, such as computational complexity and non-random patterns of missing values.

### 4.4. Elastic-Net-Based Dimension Reduction

Elastic-net regression is utilized to reduce the dimension of the impact matrix only to include indicators that significantly impact food security. To achieve an optimal outcome in the elastic-net regression, the shrinkage parameters ($\lambda_{1}$ and $\lambda_{2})$ were optimized using the *k*-*fold* cross-validation (CV) method. The CV was conducted using Statistical Package for the Social Sciences (IBM^®^-SPSS^®^ v29) software, a widely recognized tool for advanced data management and statistical analysis.

Due to the moderate sample size (94 countries) and the structure of SPSS’s elastic-net implementation, *k =* 3 was chosen to ensure sufficient data in each fold for coefficient stability. Preliminary tests with higher *k* values (e.g., 5 or 10) did not enhance model performance, supporting this choice. Similar practices have been recommended in the literature for modest datasets to manage variance and ensure convergence [80,81]. Two reduced models (M1 and M2) with dimensions (p = 15 and p = 9) were identified. However, given that the initial pool size of indicators is 25, we believe that 15 and 9 are still significant numbers. The ranges of $\lambda_{1}$ and $\lambda_{2}$ are set at (0.10 to 0.14) and (0.40 to 0.24), respectively. These narrow intervals were selected after broader preliminary tuning experiments showed that optimal performance was consistently obtained within these specific bounds. Constraining the range improved model precision and reduced unnecessary variability in parameter estimation.

Standard error (SE) is a widely used metric to assess the precision of regression models. In the context of this study, the SE reflects the average variation between the predicted prevalence of undernourishment, the % of the population (the dependent variable), and the actual observed prevalence in the population for a given combination of indicators. Based on the results presented in Table 3, one can say that M2 is a more optimized model for predicting malnutrition prevalence. The M2’s lower SE implies that its predictions exhibit less deviation from the actual prevalence data compared to M1.

Table 4 reports the reduced dimensions of the four food security categories and the regression coefficients for the remaining indicators. The “Reduction/Category, %” and “Average Reduction, %” columns display the percentage and average reduction in each category’s original number of indicators.

The percentage of model reduction significantly plays a vital role in balancing the information retained and the resulting model complexity. To understand the trade-off between complexity and information content in the food security scores, we will compare the changes in the GFSI ranking scores of the two reduced models.

### 4.5. Food Security Score Calculation

This section illustrates the calculations of the proposed food security index. However, while reducing the number of indicators may appear initially advantageous, it can have two significant consequences. First, it can hide important information regarding a country’s food security performance. For example, removing indicators such as political and social barriers to access (PASBA) or micronutrient availability (MA) could mask systemic access issues or hidden forms of malnutrition. Second, it can impact the ability of decision makers and stakeholders to understand the nature of the problem and develop effective, targeted solutions. For instance, without indicators like market access and agricultural financial services (MAAFS) or agricultural infrastructure (AIS), it becomes more difficult to distinguish whether food insecurity stems from logistical, financial, or policy-related barriers. Given that there is no specific metric for optimal dimensionality, this study will evaluate the new index utilizing the M1 model, which we believe has an acceptable dimension (*p* = 15) compared with M2 (*p* = 9) and is slightly larger than M2. However, the M2 model will be utilized later to examine the impact of the dimension reduction on the food security scores.

Also, while the regression coefficients in Table 3 provide clear insights into the direction and strength of the relationships between the indicators and malnutrition prevalence, it is essential to acknowledge that using these coefficients directly as weights remains an active area of research, especially when the relationships between indicators and response variable are not significantly linear. To avoid potential limitations associated with the regression coefficient-based weighting, this paper will utilize the two weighting approaches used by the existing GFSI: equal weighting and expert-based weighting.

**Figure 2:**
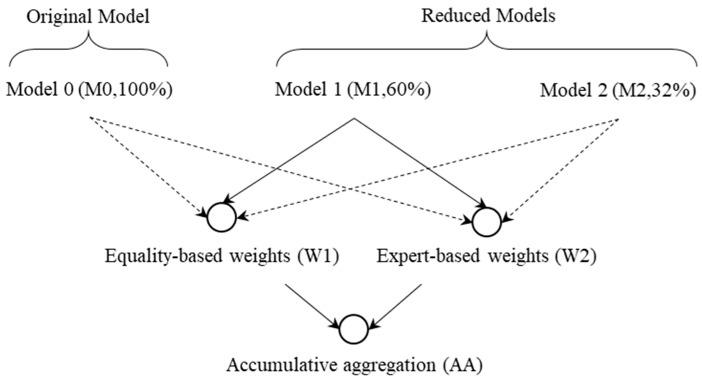
Models for calculating the elastic-net-based GFSI.

The M1 model will be examined using two different weighting approaches: equal-weighting (W1) and expert-based weighting (W2). In the following, the examined models will be referred to as M1W1AA and M1W2AA, represented in Figure 2 using solid arrows. The percentages in Figure 2 (100%, 40%, and 64%) represent the percentage of remaining indicators.

#### 4.5.1. Testing the Impact of the Weighting Method

This section will examine the impact of the weighting method on the GFSI scores and rank of the reduced models. The M1 model will be examined under two different weighting approaches: equal weighting (W1) and expert-based weighting (W2). In the following, the examined models will be referred to as M1W1AA and M1W2AA, where AA refers to accumulative aggregation.

However, as the reduced model (M1) has fewer indicators (p = 15), the GFSI will redistribute the weight of each category to the new dimension of indicators using the same ratio. For instance, if a specific category has a weight equal to 0.3 and has three dimensions, then each indicator will be assigned a weight value equal to 0.1. If this category’s dimension is reduced to two indicators, then the weight of each indicator will be reassigned as 0.15.

The food security scores using the two models (M1W1AA; M1W2AA) were calculated for 94 countries. The results indicated that these models’ means and standard deviations are {61.58; 12.54} and {63.0; 15}, respectively. Another *t*-test was conducted to investigate the significance of the difference between these models’ means of food security score. The *t*-test resulted in a *p*-value = 0.484. However, since the *p*-value ˃ α = 0.05, we fail to reject H_0_ and conclude that there is no statistically significant difference between the means of the food security scores of these models. Another rank change comparison examines how the food security scores are sensitive to the weighting method (see Table 5).

Despite the lack of statistical significance in the mean scores, the results of this comparison show that a substantial proportion of countries (80.83%) experienced changes in their food security rankings when the weighting scheme was altered from equal (W1) to expert-based (W2). Nearly half of the countries (47.87%) shifted by four or more ranks. These findings illustrate the dual implications of weighting choices. On the one hand, the expert-based method allows domain-specific priorities to shape the index, potentially giving more weight to underemphasized but strategically essential indicators. On the other hand, this approach can introduce subjectivity and increase the “judgment cost” for end-users who must interpret or justify the resulting scores. In particular, when expert weighting amplifies the influence of relatively minor indicators, rank shifts may reflect methodological decisions rather than actual performance differences, potentially complicating interpretation and reducing transparency. By contrast, equal weighting provides a neutral and easily interpretable baseline, although it may oversimplify the relative importance of indicators and ignore interdependencies. These trade-offs underscore the importance of adopting transparent, data-driven weighting strategies that balance interpretability, replicability, and policy relevance.

It is important to note that direct comparisons between these models and the original GFSI might be limited due to variations in indicator subsets and weights used by the GFSI. However, the importance of M1W1AA and M1W2AA lies in their focus on a smaller set of crucial indicators. This focus could benefit stakeholders and policymakers seeking to address food insecurity challenges and develop practical solutions.

#### 4.5.2. Testing the Impact of the Dimension Reduction

This section investigates the influence of dimension reduction on the rankings derived from the food security scores. We will compare the performances of M1W1AA and M2W1AA using the same weighting technique. Two key factors are expected to affect model performance. These are the type and number of selected indicators. As shown in Table 3, M1 and M2 share only eight indicators (PPUGP, FSNP, FSAAPC, MA, PQ, E, L, and S). Notably, M1 utilizes eight additional indicators not included in M2. This difference in indicator selection might lead to significant variations in the resulting food security scores and rankings.

The food security scores were calculated for 94 countries using the two models. The analysis revealed that the means and standard deviations for the food security scores of these models were (61.58; 12.54) and (66.89; 14.25), respectively. A two-sample *t*-test was conducted to statistically assess the significance of the difference in the mean food security scores between the M1W1AA and M2W1AA models.

The *t*-test results showed that the *p*-value = 0.07 (˃α= 0.05). This leads to the conclusion that there is no significant difference between the means of the food security scores of M1W1AA and M2W1AA. In other words, there is no strong evidence of a substantial difference between the average food security scores produced by the two models. This finding can be attributed to two key factors. First, applying dimension reduction techniques can lead to different sets of indicators being included within the same food security category across various models. Second, even if the weight assigned to a specific category remains equal across all its indicators, variations in the indicator sets used by the compared models can still result in different weights being assigned, even to common indicators. In other words, the weight of a particular indicator on the food security scores and ranks can vary significantly between models that utilize different dimensions. However, unlike the PCA and FA, which aim to capture overall data variability through linear transformation reduction, elastic-net regression prioritizes selecting the most influential features. Consequently, this finding may not necessarily generalize to other dimensionality reduction techniques, which often prioritize capturing variance rather than feature selection.

## 5. Two-Step Cluster Analysis

The two-step cluster analysis represents a critical contribution to this paper. By applying this method, we can comprehensively understand the relationship between food security scores and food system characteristics. The two-step approach can help identify clusters of countries with comparable food security performance and identify key characteristics that most significantly contribute to a country’s performance.

The two-step cluster is a data clustering technique that can handle large sets of data, and it uses both categorical and continuous variables to create data clusters. The objective is to group similar observations based on data attributes. Utilizing two-step clustering can help cluster countries not just on one basis but with different parameters, which allows us to understand the nature of clusters better and, hence, understand the countries’ food security performance.

This two-step clustering will be applied to the M1W1AA model, using inputs of food security scores, food system type, and economic state of the countries involved. The last two inputs represent the characteristics of the food system. The food system type involves five categories: emerging and diversifying, industrial and consolidated, informal and expanding, modernizing and formalizing, and rural and traditional [82]. The economic state is classified into four categories based on the gross national income (GNI) [83]. These are low-income, low–middle–income, upper–middle–income, and high-income.

### 5.1. Step One: Food Security Score-Based Clustering

**Figure 3:**
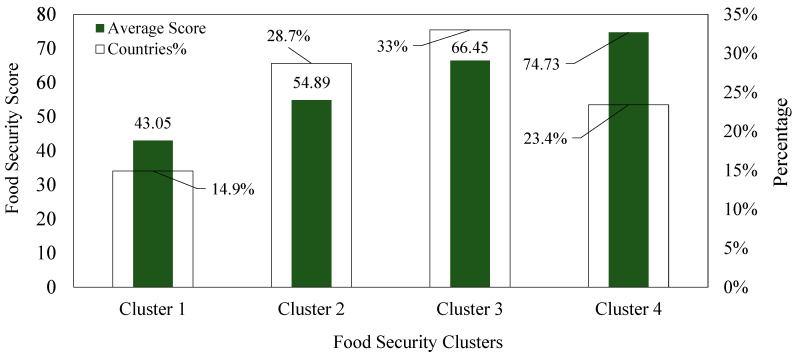
Food security clusters using the M1W1AA model.

In the first step, countries are grouped based on their food security scores to identify clusters with similar performance levels. As shown in Figure 3, 14.9% of countries fall into the low-performing cluster (Cluster 1), with an average score of 43.05. Meanwhile, 23.4% belong to the high-performing cluster (Cluster 4), with a mean score of 73.73. The remaining 61.7% are distributed across Clusters 2 and 3, representing average-performing countries, with scores of 54.89 and 66.45, respectively. While this initial clustering helps categorize countries by performance, it does not reveal why such differences exist. Understanding the underlying food system characteristics is essential for developing targeted strategies and learning from better-performing peers. This motivation drives the second step of the analysis.

### 5.2. Step Two: Characteristic-Based Clustering

The second step assesses the relationship between food system type, economic status, and food security outcomes. Based on these two characteristics, the clusters identified in the previous step will be analyzed to understand how the food system type and economic status may influence food security scores. This step explores how different combinations of food system characteristics and economic status contribute to variations in food security outcomes across the clusters.

**Figure 4:**
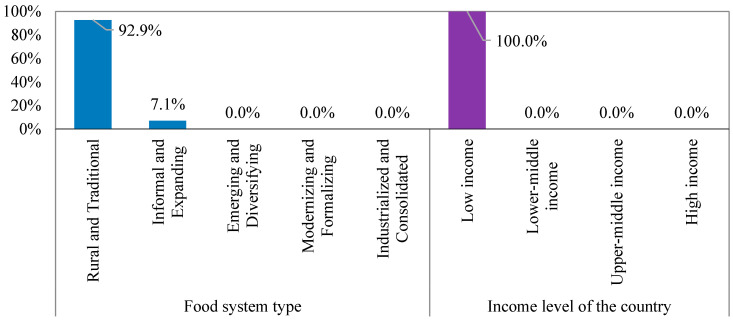
Food system characteristic-based grouping of Cluster 1.

Figure 4 shows that 92.9% of the countries in Cluster 1 adopt rural and traditional food systems. These systems often face challenges, inefficient transportation networks, financial shortages, and a lack of technological advancement, making them less secure than other systems. However, while adopting rural and traditional food systems in low-income countries may offer some environmental advantages, it might limit the capacity to maintain food security.

**Figure 5:**
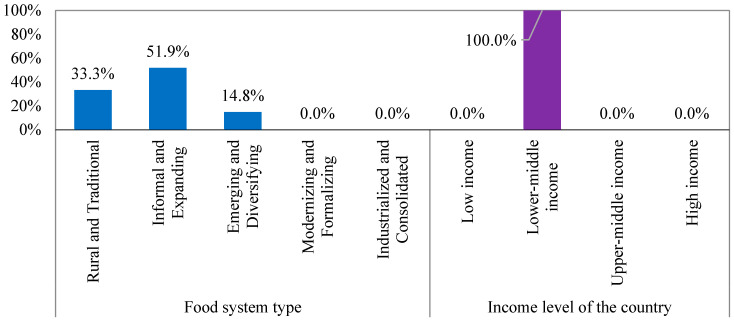
Food system characteristic-based grouping of Cluster 2.

Figure 5 reveals that 51.9% of the countries in Cluster 2 adopt informal and expanding food systems. The remaining 48.1% of the countries adopt either emerging and diversifying or rural and traditional food systems. Notably, although 33.3% of the countries in this cluster adopted rural and conventional food systems, such as Tanzania, Kenya, and Bangladesh, these countries demonstrated high security scores compared with Cluster 1. The economic strength of these countries likely explains this finding. All the countries in Cluster 2 have lower–middle income levels, which offer limited but essential resources to mitigate some food security challenges associated with rural and traditional food systems. This underscores the crucial role of economic status in shaping food security outcomes.

**Figure 6:**
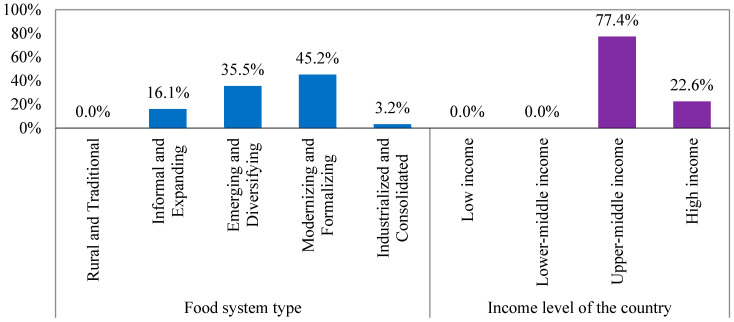
Food system characteristic-based grouping of Cluster 3.

Figure 6 shows that 45.2% of the countries within Cluster 3 adopt the modernizing and formalizing food system type, while 35.5% adopt the emerging and diversifying food system type. The economic situation regarding the countries’ GDP in this cluster varies between upper–middle (77.4%) and high income (22.6%). The rise in the income level of the countries in Cluster 3 significantly contributes to improving the food systems of these countries, such as Indonesia, Azerbaijan, and Thailand.

**Figure 7:**
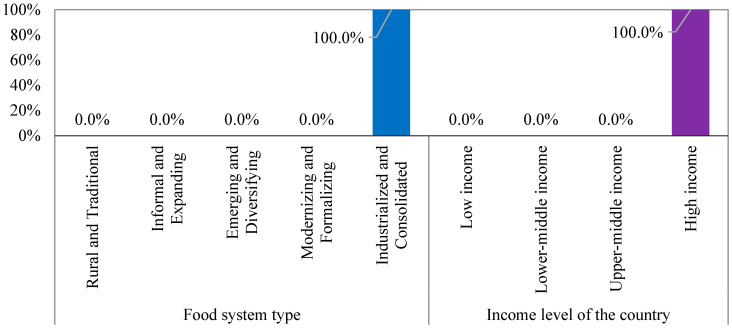
Food system characteristic-based grouping of Cluster 4.

Figure 7 shows that 100% of the countries in Cluster 4 adopt industrialized and consolidated food system types, including Switzerland, Germany, and Finland. These systems are well known for utilizing advanced technology and automation to maximize their productivity and efficiency, which are important for maintaining economic growth and ensuring self-sufficiency in food and nutrition. The results also show that 100% of these countries are classified as high-income countries. This finding is unsurprising, as many jobs across various sectors, from manufacturing and engineering to logistics and transportation, created by these systems likely contribute to their economic status.

The two-step cluster analysis unveiled a significant correlation between food system type, economic status, and food security level. This revelation is crucial for understanding the dynamics of food security. While industrialized and consolidated food systems have demonstrated adequate food security levels, rural and traditional food systems face serious challenges. This is particularly true for countries with low income levels, underscoring the importance of our research in shedding light on these critical issues.

Most of the existing food security analyses use traditional score-based ranking, which does not consider the characteristics of the food system. Scores provide a single numeric value, which can oversimplify the multifaceted nature of food security. They may not capture underlying issues and may even ignore context-specific factors like cultural food preferences, regional disparities, and the impact of local policies, which are crucial for a holistic understanding of food security. They also lack nuance in policymaking, wherein relying solely on scores may lead to generic policy recommendations that may be ineffective for all regions or populations. Different areas might have similar scores but require distinct interventions due to their unique challenges. For instance, despite similar food security scores, i.e., 76, Poland and China require different food security strategies due to distinct challenges, as they have different income levels and types of food systems, too. Poland, a high-income country with an aging population and EU policy constraints, should focus on agricultural innovation, rural development, and adapting EU policies for competitiveness. Meanwhile, China, an upper–middle-income nation facing rapid urbanization, environmental degradation, and food safety issues, must prioritize sustainable farming, stringent food safety regulations, and optimizing supply chains. These strategies reflect each country’s unique socioeconomic and environmental contexts, highlighting the need for tailored approaches to achieve food security. While different areas having similar scores may need different interventions because of their unique challenges, vice versa also holds true. Kenya was awarded a food security score of 47.4, while Cambodia could only manage to secure a food security score 57.7. Both countries have rural and traditional food systems and belong to the same “lower–middle income” category. Both countries need investments in rural infrastructure and access to markets. Despite Cambodia’s higher score, both face challenges related to rural poverty, underdeveloped infrastructure, and limited market access. Interventions in road building, market access improvements, and rural development programs would benefit both.

To assess the external relevance of the identified clusters, we compared our results with country classifications from the 2019 Global Hunger Index (GHI) [84], a widely recognized indicator of food insecurity severity. While our clustering framework was designed to capture structural characteristics, namely food system typologies and economic status, rather than outcome measures, this comparative analysis provides useful context.

The comparison revealed a notable alignment between our clusters and the GHI categories. Countries grouped into Cluster 1, characterized by rural and traditional food systems and low-income status, predominantly fall under the “Serious” or “Alarming” GHI categories (e.g., Yemen, Madagascar, and Chad). In contrast, Cluster 4, composed mainly of high-income countries with industrialized and consolidated food systems, corresponds closely with countries categorized as “Low” in the GHI (e.g., Germany, Canada, and Australia). Intermediate clusters (Clusters 2 and 3) include countries with lower–middle or upper–middle income levels and a broader diversity of food system structures, reflecting a wider spread across the “Moderate” to “Low” GHI categories.

This alignment suggests that while our typology does not directly mirror score-based indices, it captures meaningful structural dimensions correlating with food security outcomes. The results support the view that food insecurity is not only a matter of outcomes but is also deeply rooted in systemic configurations, reinforcing the potential of structural clustering approaches as complementary tools to traditional classification systems.

Relying solely on score-based rankings may result in misguided prioritization by policymakers, as these scores often lack the nuance required to target specific needs. Identifying the underlying drivers of food insecurity becomes challenging without incorporating structural characteristics such as the food system type, income level, infrastructure, market access, etc. Structural clustering, thus, presents a more comprehensive and actionable framework for designing effective, context-specific interventions.

## 6. Conclusions and Remarks

This study presented a new food security assessment approach that combines two well-established methods, feature selection and two-step clustering, to address some of the limitations of its existing counterparts and provide decision makers and stakeholders with systematic procedures for making informed decisions and supporting food system management. The new approach’s mathematical and operational procedures were assessed using the EIU dataset of 94 food-producing countries. Below are valuable findings and remarks extracted from this study.

The analysis reveals the influence of the food system typology and economic state on the food security levels of the underlying countries. Countries with industrialized and consolidated food systems, typically high-income economies such as Switzerland and Germany, consistently demonstrate the highest levels of food security. In contrast, countries with rural and traditional food systems and low-income status exhibited significantly lower performance. For instance, 92.9% of the countries in Cluster 1 adopted rural and traditional food systems and faced numerous challenges, including limited infrastructure and technological capabilities.

The cluster-based output format of the proposed method enhances the interpretability and practical utility of food security assessments. It enables policymakers to link aggregated scores with underlying country characteristics and context-specific design interventions. For example, countries in clusters dominated by rural and traditional food systems could benefit from policies to improve transportation infrastructure, expand access to agricultural finance, and foster agro-technological innovation. Development partners might prioritize investments in areas such as farmer training, mobile extension services, and climate-resilient crop systems. Conversely, in countries with industrialized food systems but persistent food insecurity, policy efforts could focus on improving food distribution equity, supporting smallholder inclusion, or reducing food waste. Tailoring interventions based on cluster identity facilitates more efficient resource allocation and ensures that policy responses are context-sensitive and impactful. Importantly, the study highlights that similar food security scores can mask different underlying realities. For instance, although Kenya and Cambodia received different food security scores (47.4 and 57.7, respectively), both share the same income level and food system type, indicating similar policy needs. Conversely, despite having comparable scores, countries like Poland and China require distinct strategies due to differences in their economic profiles and food system typologies. These examples underscore the added value of the clustering approach in uncovering policy-relevant patterns that a single score cannot.

While clustering enhances interpretability and supports tailored policy design, it is essential to acknowledge that typologies may not capture the full complexity of country-specific contexts. Over-reliance on aggregated cluster identities risks overlooking unique socio-political, cultural, or institutional dynamics influencing food security. Therefore, while the clustering framework provides a helpful starting point for targeted intervention design, policymakers are encouraged to complement it with country-level diagnostics and stakeholder engagement to ensure relevance and effectiveness.

The new approach has shown appropriate applicability in assessing food security. By adjusting the two shrinkage parameters of the elastic-net regression, the practitioner can examine and analyze food security performance using different subsets of indicators and dimensions. This feature can help to investigate how the inclusion of specific single or multiple groups of indicators influences the overall performance of food system security. Although the proposed approach effectively captures food security performance, some limitations should be acknowledged. First, the analysis is based solely on cross-sectional data from GFSI 2019, which limits the ability to track changes or trends over time. Expanding to multi-year datasets would allow for a more dynamic understanding. Second, relying on a single data source may constrain indicator diversity and coverage; incorporating other datasets could offer a more comprehensive view. Third, elastic-net regression assumes linear relationships between predictors and outcomes, which may not fully capture the complex nature of food systems. This limitation could be addressed using non-linear models such as random forests or neural networks. Although missing data were handled through imputation, some sensitivity to outlier handling and imputation choices persists, and future work should systematically compare alternative methods. Finally, the clustering relied on only two country-level features; involving experts to guide the inclusion of more relevant characteristics could enhance the contextual relevance of the groupings. While this study used country-level data for cross-sectional analysis, future work could test the framework at the subnational level to better capture local disparities. Moreover, incorporating additional dimensions such as food system resilience or environmental stressors could enhance the model’s capacity to inform targeted, region-specific interventions. Additionally, while the method may not be immediately transferable to regions with limited data, it provides a flexible framework that can be adapted over time. In low-data contexts, the methodology can prioritize available key indicators and gradually improve its robustness as more data becomes accessible, making it applicable to a broader range of settings.

## Figures and Tables

**Table 1:** Structure of food security categories.

Category	No.	Indicators	Symbol
Affordability (AFF)	1	Change in the food costs	CFC
	2	Proportion of population under the global poverty line	PPUGP
	3	Inequality-adjusted income index	IAII
	4	Agricultural import tariffs	AIT
	5	Food safety net programs	FSNP
	6	Market access and agricultural financial services	MAAFS
Availability (AVA)	7	Sufficiency of supply	SOS
	8	Agricultural research and development	ARAD
	9	Agricultural infrastructure	AIS
	10	Volatility of agricultural production	VOAP
	11	Political and social barriers to access	PASBA
	12	Food loss	FL
	13	Food security and access policy commitments	FSAAPC
Quality and safety (QAS)	14	Dietary diversity	DD
	15	Nutritional standards	NS
	16	Micronutrient availability	MA
	17	Protein quality	PQ
	18	Food safety	FS
Natural resources and resilience (NR&R)	19	Exposure	E
	20	Water	W
	21	Land	L
	22	Oceans, rivers, and lakes	ORL
	23	Sensitivity	S
	24	Political commitment to adoption	PCTA
	25	Demographic stress	DS

**Table 2:** Pearson correlation coefficients between the indicators under the Natural Resources and Resilience category and GFSI.

Variable	GFSI	NR&R	E	W	L	ORL	S	PCTA	DS
GFSI	1.000	0.684	−0.036	0.531	0.315	−0.352	0.243	0.535	0.803
NR&R	0.684	1.000	0.135	0.694	0.428	−0.158	0.399	0.796	0.571
E	−0.036	0.135	1.000	−0.061	0.064	−0.083	−0.016	−0.028	−0.199
W	0.531	0.694	−0.061	1.000	0.271	−0.098	0.038	0.390	0.385
L	0.315	0.428	0.064	0.271	1.000	−0.051	−0.075	0.094	0.227
ORL	−0.352	−0.158	−0.083	−0.098	−0.051	1.000	−0.386	−0.306	−0.401
S	0.243	0.399	−0.016	0.038	−0.075	−0.386	1.000	0.317	0.426
PCTA	0.535	0.796	−0.028	0.390	0.094	−0.306	0.317	1.000	0.406
DS	0.803	0.571	−0.199	0.385	0.227	−0.401	0.426	0.406	1.000

Note: For the abbreviation details of variables, please refer to Table 1.

**Table 3:** Food security rank comparison based on the imputation method.

Rank Change	Number of Countries	Percentage, %
No change	70	75
+/− 1 rank	17	18
+/− 2 ranks	6	6
+/− 3 ranks	1	1
+/− ≥4 ranks	0	0

**Table 4:** Elastic-net regression coefficients and reduced dimension.

Category	Indicator	Coefficient Estimators		Reduction, %		Average Reduction, %
		M1	M2	M1	M2	
Affordability	CFC	-		50.00	66.67	58.34
	PPUGP	0.06	0.004			
	AIT	−0.02	-			
	FSNP	0.45	0.35			
Availability	SOS	−0.03	-	57.14	70.14	63.64
	ARAD	−0.05	-			
	PASBA	-	−0.003			
	FL	-	-			
	FSAAPC	0.09	0.15			
Quality and safety	DD	-	-	40.00	60.00	50.00
	NS	−0.02	-			
	MA	−0.13	−0.07			
	PQ	0.05	0.01			
	FS	-	-			
Natural resources and resilience	E	0.06	0.08	28.57	57.14	42.86
	L	−0.34	−0.27			
	ORL	0.02	-			
	S	−0.06	−0.01			
	PCTA	−0.05	-			
	DS	0.06	-			

**Table 5:** Food security rank comparison based on the weighting method.

Rank Change	Number of Countries	Percentage, %
No change	18	19.15
+/− 1 rank	9	9.57
+/− 2 ranks	14	14.89
+/− 3 ranks	8	8.51
+/− ≥4 ranks	45	47.87

## Data Availability

The original contributions presented in this study are included in the article. Further inquiries can be directed to the corresponding author.

## Abbreviations

**Acronym**	**Definition**
IQR	Interquartile Range
AHP	Analytical Hierarchy Process
CV	Cross-Validation
DEA	Data Envelope Analysis
EIU	Economist Intelligence Unit
FA	Factor Analysis
FAO	Food and Agriculture Organization
FIES	Food Insecurity Experience Scale
GFSI	Global Food Security Index
GHI	Global Hunger Index
GNI	Gross national income
IFPRI	International Food Policy Research Institute
LASSO	Least Absolute Squared Shrinkage Operator
NR&R	Natural resources and resilience
PCA	Principal Component Analysis
SE	Standard error
SPSS	Statistical Package for the Social Sciences
TOPSIS	Technique for Order of Preference by Similarity to the Ideal Solution

List of Mathematical Notations/Abbreviations

**Symbol**	**Description**
y	The dependent variable for the elastic-net regression
x	The independent variables for the elastic-net regression
$\beta_{j}$	The coefficients of the regression model
ε	Normally distributed random error term
$P_{\lambda }(\beta )$	Penalty function
r	Pearson’s correlation coefficient
$l_{1}$ and $l_{2}$	Penalty functions 1 and 2
$\lambda_{1}$ and $\lambda_{2}$	Shrinkage parameters
$x_{ij}$	The original score of the ith country under the jth indicator
M_*m**a**x*,*j*_ and M_*m**i**n*,*j*_	Represent the maximum and minimum scores of the *j*th indicator

## Appendix A

**Table A1:** Two-sided t-test: two-sample Z-score.

Statistic	Value
Difference ($\mu_{pre}-\mu_{post})$	−0.630
Observed *t*	−0.658
Critical |t|	2.074
Degrees of freedom	22
*p*-value (two-tailed)	0.517
Significance level (α)	0.05
